# IL-35 alleviates inflammation progression in a rat model of diabetic neuropathic pain via inhibition of JNK signaling

**DOI:** 10.1186/s12950-019-0217-z

**Published:** 2019-07-23

**Authors:** Yinghai Jiang, Jing Wang, Haiqin Li, Lingjie Xia

**Affiliations:** Pain Department, Henan Provincial People’s Hospital, No. 7 Weiwu Road, Zhengzhou, 450003 Henan China

**Keywords:** Diabetic neuropathic pain, IL-35, Inflammation, JNK signaling

## Abstract

**Background:**

Emerging evidence has demonstrated that inflammation is involved in the occurrence and development of diabetic neuropathic pain (DNP). The anti-inflammatory property of interleukin (IL)-35 makes it a promising candidate to block the pain perception. The present study was undertaken to investigate whether IL-35 could attenuate DNP in streptozotocin (STZ)-induced rat model and its potential mechanism.

**Methods:**

The rat model of DNP was established by a single STZ injection followed by measurements of fasting blood glucose and insulin. Fourteen days after STZ injection, DNP rats were intrathecally injected with IL-35, c-Jun N-terminal kinase (JNK) inhibitor or activator or dimethylsulfoxide (DMSO) as vehicle control, respectively. The mechanical allodynia was assayed to evaluate the therapeutic effect of IL-35. In mechanism study, the serum and protein levels of inflammatory cytokines using ELISA and western blotting and the activation of JNK signaling were further evaluated by quantitative reverse transcription PCR (qRT-PCR). Histopathologic changes were evaluated by Nissl staining. Apoptosis was examined using TUNEL staining.

**Results:**

DNP rats exhibited increased fasting blood glucose and insulin levels and reduced insulin sensitivity index (ISI). Intrathecal injection of IL-35 reduced accumulation of pro-inflammatory cytokines in the spinal cord of DNP rats. Furthermore, IL-35 displayed anti-inflammatory and anti-apoptotic effects via inhibition of JNK pathway.

**Conclusion:**

IL-35 treatment mitigated DNP via downregulating JNK signaling pathway.

## Introduction

Diabetes is a common chronic metabolic disease threatening human health. With the improvement of people’s living standard, the aggravation of ageing population and the alteration of people’s life style, the prevalence of diabetes is gradually increasing [1]. Diabetic neuropathic pain (DNP) is one of the most common chronic complications of diabetes, which is characterized by spontaneous pain, hyperalgesia, and allodynia, affecting more than 50% of patients living with diabetes [2]. Currently, the pathogenesis of DNP is not fully understood. Several lines of evidence suggest that inflammation, metabolic disorders and oxidative stress are the principal determinants for its development [3]. Until now, it is difficult for any single treatment to achieve satisfactory efficacy.

Mitogen-activated protein kinase (MAPK) cascade is one of the important signal transduction systems in response to a wide variety of extracellular stimuli, which is involved in regulating diverse cellular functions such as cell growth, proliferation, apoptosis, and differentiation and many pathophysiological processes including inflammation and wound healing [4]. There are mainly three MAPK signaling pathways in mammalian cells, including extracellular signal-regulated protein kinase (ERK), c-Jun N-terminal kinase (JNK) and p38-MAPK, which are known to participate in the generation of pain hypersensitivity [5]. It is noteworthy that JNK-c-Jun signaling pathway is essential for chronic inflammatory pain [6]. Studies have shown that JNK could be activated by pro-inflammatory cytokines, thereby serving as a crucial mediator of inflammatory pain sensitization via the activation of microglia and astrocytes [7]. However, the inhibition of JNK signaling pathway could effectively relieve inflammatory and neuropathic pain in different animal models [8]. Consequently, JNK pathway might be used as a molecular target for treatment of neurodegeneration and chronic pain in patients with neurodegenerative diseases such as spinal cord injury and diabetic neuropathy.

Interleukin (IL)-35 is a heterodimeric cytokine composed of IL-12p35 and Epstein-Barr virus-induced gene 3 (EBI3) subunits with anti-inflammatory and immunosuppressive capacities and plays an important role in various diseases. Yan et al. [9] demonstrated that serum and vitreous levels of IL-35 were significantly decreased in patients with proliferative diabetic retinopathy (PDR). Lower serum levels of IL-35 were also detected in patients with gestational diabetes mellitus (GDM) and preeclampsia (PE) [10]. A large quantity of evidence has revealed that IL-35 could inhibit tumor necrosis factor (TNF)-α-induced activation of nuclear factor (NF)-κB via the signal transducer and activator of transcription 1 (STAT1) phosphorylation and suppressed the phosphorylation of MAPK family including JNK, ERK, and p38 activated by lipopolysaccharide (LPS) [11, 12].

Therefore, in the present study, we investigated that IL-35 might affect streptozotocin (STZ)-mediated DNP. Specifically, it was hypothesized that IL-35 should inhibit the expression of inflammatory cytokines by inhibiting the activation of JNK signaling pathway, thereby ameliorating DNP in rats.

## Materials and methods

### Animals

All animal experiments were performed in accordance with the guidelines for Laboratory Animal Care and Use of Henan provincial people’s hospital. Specific pathogen-free (SPF) grade male Sprague-Dawley (SD) rats weighing 200–220 g which were purchased from the Experimental Animal Center of Henan provincial people’s hospital were housed individually in an air-conditioned room maintained at a temperature of 22 °C ± 2 °C and 12−/12-h light/dark cycle and were allowed to eat and drink ad libitum. All mice received adaptive feeding for 3 days prior to the formal experiment. All animal procedures and protocols were approved by the Institutional Animal Care and Use Committee of Henan provincial people’s hospital.

### DNP model induction

For induction of DNP, SD rats were fed a high-sugar and high-fat diet containing 67% commercial rodent diet (Certified Rodent Diet 5002; PMI Feeds, Inc., Richmond, IN, USA), 10% lard, 20% sucrose, 2% cholesterol, and 1% sodium cholate with free access to water. Fasting plasma glucose levels and fasting insulin levels were measured by routine methods, and insulin sensitivity index (ISI) was calculated at 0 and 8 weeks of feeding. After 8 weeks of feeding, the fasting insulin concentration were significantly elevated, while the ISI decreased, which suggested that insulin resistance appeared, the rats were then intraperitoneally administrated with 35 mg/kg STZ (Sigma-Aldrich, St. Louis, MO, USA) dissolved in 0.9% sterile saline to induce diabetes. Three days later, type I diabetes mellitus was confirmed in STZ-injected rats by measuring fasting plasma glucose concentrations in blood samples obtained from the tail vein. Only rats with blood glucose concentrations greater than 16.7 mmol/L were further used in this study. Two weeks after STZ injection, the eligible rats were again measured for mechanical withdrawal thresholds (MWT), and the rats with pain threshold ≤85% base value were selected for the rat model of type I diabetes neuropathic pain.

### Experimental design

A total of 36 SD rats were randomly divided into 6 groups after 72 h of feeding adaptation (*n* = 6 per group), namely, Control group, the normal diet group, DNP group, DNP rats injected with normal saline, DNP + vehicle group, DNP rats injected with dimethylsulfoxide (DMSO), SP group, DNP rats injected with JNK inhibitor SP600125, IL-35 group, DNP rats injected with recombinant IL-35 and IL-35 + Ani group, DNP rats injected with recombinant IL-35 and JNK activator anisomycin. Briefly, DNP rats were anesthetized with 2% halothane. The PE-10 catheters (1 cm) were inserted into the subarachnoid space through L4–5 intervertebral space. After 3 days recovery following cannulation, the rats were intrathecally administrated with 10 μL normal saline, 10 μL DMSO (Sigma-Aldrich), 5 μL SP600125 (Celgene, Inc., San Diego, CA, USA), 5 μg/kg IL-35 recombinant protein (Sino Biological Inc., Beijing, China) and IL-35 recombinant protein plus anisomycin (10 mg/kg; Sigma-Aldrich) daily for continuous 14 days. The MWT and thermal withdrawl latency (TWL) were measured at 1, 3, 7 and 14 days after subarachnoid injection. Blood samples were collected and the fourth to sixth lumbar segment of the spinal cord were removed after 14 days of injection for the following experiments.

### Blood glucose detection

Plasma glucose levels were measured using Glucotrend (Roche Diagnostics, Mannheim, Germany), according to the supplier’s instructions.

### Determination of insulin and ISI

After standing at room temperature for 5 min, the tail vein blood samples were centrifuged at 4000 rpm for 20 min at 4 °C. The supernatants were collected, which were used to measure insulin concentration by sandwich ELISA kit (Sigma-Aldrich). The ISI was then calculated using the following formula: ISI = 1/(fasting glucose × fasting insulin).

### Mechanical allodynia assessment

The behavioral testing was conducted between 8:00 AM and 17:00 PM. To quantify mechanical sensitivity of the hind paw, rats were placed in individual plastic boxes (22 cm × 22 cm × 22 cm) on a mesh floor (1 cm × 1 cm) and allowed to acclimate for 15 min. IITC 2390 series electronic von Frey tactile pain measurement instrument (Stoelting, Wood Dale, IL, USA) was perpendicularly applied to the plantar surface of the hind paw with sufficient force. The intensity of the stimulus was measured when the rats lifted or licked their feet. Each trial was repeated 5 times at approximately 10s intervals, and the mean value was used as the force to produce withdrawal responses.

The rats were placed on a glass box with 3 mm thick, thermal radiation was applied to the hind toe using tail flick apparatus (IITC Inc., Woodland Hills, CA, USA), and the time from irradiation to paw withdrawal was recorded. The feet of each rat were tested 5 times, with 5 min time intervals. The values of the last three times were averaged as TWL.

### ELISA for cytokine measurement

The serum levels of inflammatory cytokines including IL-35, IL-1β, IL-6, TNF-α, and IL-10 were determined using the quantikine enzyme-linked immunosorbent assay kit (R&D Systems, Minneapolis, MN, USA) following the manufacturer’s instructions. Each blood sample was tested in duplicate.

### Western blot

Prior to western blotting, frozen tissue specimens were incubated in lysis buffer for 30 min before being centrifuged at 12,000 rpm at 4 °C for 20 min. The supernatant was then collected, and the protein concentration was determined using the bicinchoninic acid method. Equal amounts of total protein (35 μg) were resolved by 10% SDS polyacrylamide gel electrophoresis (SDS-PAGE), transferred onto polyvinylidene fluoride (PVDF) membranes and blocked in 5% skim milk in tris buffered saline tween (TBST) at 4 °C overnight. Blots were probed with EBI3 (1:500 dilution, Imgenex, San Diego, CA, USA), JNK and phospho-JNK antibodies (1:1000 dilution; Cell Signaling Technology, Boston, MA, USA), then incubated with horseradish-peroxidase-coupled antibodies (1:2000; Peprotech, Rocky Hill, NJ, USA) at 37 °C for 2 h and visualized on a Molecular Imager ChemiDoc XRS System (Bio-Rad Laboratories, Hercules, CA, USA) using an ECL Plus Western Blotting Substrate (Thermo Scientific, Shanghai, China). In addition, β-actin (Sigma-Aldrich) was served as an internal control.

### QRT-PCR

Total RNA was extracted from spinal cord tissues using Trizol reagent (Invitrogen) following the manufacturer’s protocol and reversely transcribed to cDNA using Primer-Script TM one step RT-PCR kit (Takara, Shiga, Japan) for the reverse transcription of mRNAs. The SYBR Green I real time PCR kit (CoWin Bioscience Co., Beijing, China) was used to amplify targets on an ABI 7500 Real-Time PCR system (Applied Biosystems, Carlsbad, CA, USA) and calculated by the 2^−ΔΔCt^ method. The relative expressions of IL-1β, IL-6, TNF-α, and IL-10 were normalized to GAPDH expression.

### Nissl staining

Spinal cord segments were fixed in 4% paraformaldehyde for 24 h and embedded in paraffin for transverse paraffin sections. The paraffin sections (4 μm thick) were incubated in 1% Cresyl violet for Nissl staining and observed under a light microscope (Olympus, Tokyo, Japan). The Nissl positive cells were automatically counted at 5 randomly selected fields.

### Apoptosis detection

Apoptotic cells in the spinal cord tissues were detected using terminal deoxynucleotidyl transferase-mediated dUTP nick end labeling (TUNEL) staining (Roche Diagnostics, Mannheim, Germany) according to the manufacturer’s instructions. Paraffin-embedded sections were deparaffinized with xylene, rehydrated with graded ethanol and treated with 2% hydrogen peroxide in phosphate-buffered saline (PBS) at room temperature for 5 min to block the endogenous peroxidase activity. After washed with PBS, sections were incubated with terminal deoxynucleotidyl transferase enzyme and TUNEL reaction mixture for 1 h at in the dark. Slides were counterstained with 0.05% diaminobenzine (DAB) and counted under a microscope (Olympus).

### Statistical analysis

Data were expressed as mean ± standard deviation (SD) and were analyzed using SPSS 22.0 (IBM, Armonk, NY, USA). Two or more data sets were compared using Student’s *t*-test or one-way analysis of variance, with *P* < 0.05 being considered statistically significant.

## Results

### Fasting plasma glucose and insulin levels were increased while ISI was reduced in DNP rats

As shown in Table 1, compared to the control group, the concentration of serum glucose in DNP group was significantly elevated after 8 weeks of feeding with high-sugar and high-fat diet (*P* < 0.05), but was lower than the diagnostic criteria for diabetes (> 16.7 mmol/L). In addition, DNP rats displayed higher insulin levels and lower ISI compared to the control group (*P* < 0.05).

**Table 1:** Comparison of blood glucose, insulin and ISI (mean ± SD, *n* = 6)

Variables	Time	Control	DNP
Blood glucose (mmol/L)	0 week	3.65 ± 0.35	3.95 ± 0.40
	8 week	4.11 ± 0.40	7.44 ± 0.9^*^
	STZ 3 days	5.88 ± 0.69	20.55 ± 2.99^*^
Insulin (mU/l)	0 week	7.85 ± 0.88	8.15 ± 0.87
	8 week	7.15 ± 1.05	13.01 ± 2.56^*^
ISI	0 week	−1.45 ± 0.15	−1.50 ± 0.18
	8 week	−1.46 ± 0.20	−2.04 ± 0.25^*^

^*^P < 0.05 vs. control. *ISI* insulin sensitivity index

### IL-35 alleviated DNP-induced inflammation in vivo

**Fig. 1:**
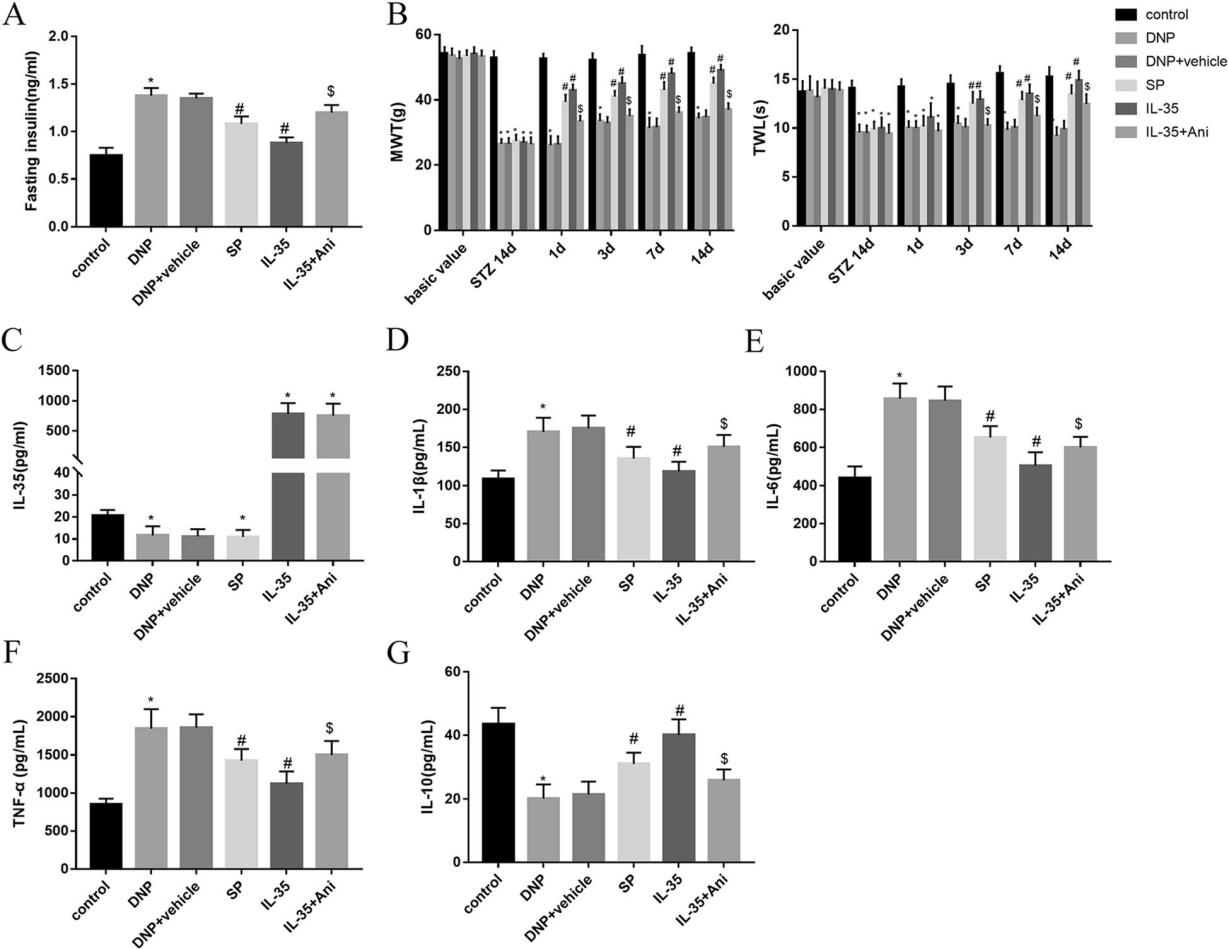
Effect of IL-35 on inflammation in a rat model of DNP. The fasting insulin concentration using ELISA assay (**a**), Changes in MWT and TWL (**b**) in baseline, after 14 days of intraperitoneal injection with STZ and after administrating different drugs for 1, 3, 7 and 14 days and serum levels of IL-35 (**c**), IL-1β (**d**), IL-6 (**e**) and TNF-α (**f**) and IL-10 (**g**) in the groups of Control, DNP, DNP + vehicle, SP, IL-35 and IL-35 + Ani. ^*^P < 0.05 vs. the control group; ^#^*P* < 0.05 vs. the DNP + vehicle group; ^$^*P* < 0.05 vs. the IL-35 group. *n* = 6 per group

Compared with the DNP group, intrathecal injection of SP600125 and IL-35 recombinant protein markedly decreased the fasting insulin levels, and treatment with JNK activator anisomycin could partially revert insulin levels (Fig. 1a; *P* < 0.05). No significant differences were observed in the basic value of MWT and TWL among all groups. However, compared with the control group, the MWT and TWL in the other groups were significantly decreased after 14 days of intraperitoneal injection of STZ. DNP rats also exhibited lower MWT and TWL as relative to the control group (*P* < 0.05). Conversely, the values of the MWT and TWL were increased at 1, 3, 7 and 14 days after administrating SP600125 and recombinant IL-35 into subarachnoid space in DNP group. At the end of the 2rd week, MWT and TWL were significantly lower in IL-35 + Ani groups than in IL-35 group (Fig. 1b; *P* < 0.05). Serum IL-35 levels were downregulated in DNP and SP group, but were highly expressed in IL-35 and IL-35 + Ani group as relative to the control group (Fig. 1c; *P* < 0.05). Moreover, we found that the levels of three pro-inflammatory factors IL-1β (Fig. 1d; *P* < 0.05), IL-6 (Fig. 1e; *P* < 0.05) and TNF-α (Fig. 1f; *P* < 0.05) in rat serum were also significantly increased, while the production of anti-inflammatory cytokine IL-10 (Fig. 1g; *P* < 0.05) was reduced in DNP group. Intrathecal injection of SP600125 or IL-35 recombinant protein markedly reduced the levels of IL-1β (Fig. 1d; *P* < 0.05), IL-6 (Fig. 1e; *P* < 0.05) and TNF-α (Fig. 1f; *P* < 0.05) and enhanced the levels of IL-10 (Fig. 1g; *P* < 0.05). In contrast, anisomycin administration partly overturned the anti-inflammatory effect of IL-35. Collectively, these findings elucidated that IL-35 treatment inhibited inflammatory response in a rat model of DNP.

### IL-35 ameliorated DNP via inhibition of JNK signaling pathway in vivo

**Fig. 2:**
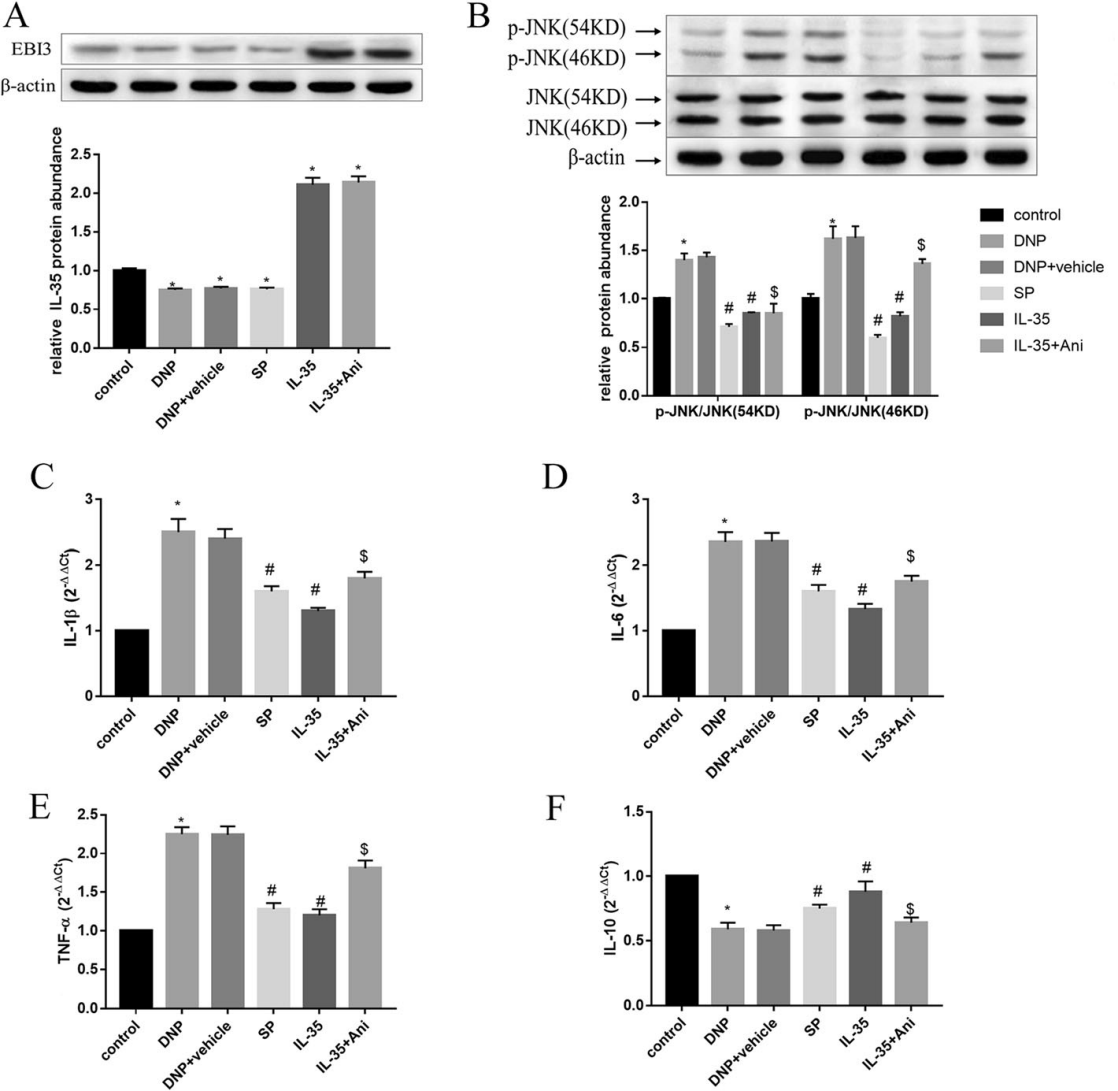
A possible mechanism for IL-35-mediated anti-inflammatory effect. The protein levels of IL-35 (**a**), JNK and p-JNK (**b**) using western blotting, and the mRNA expression levels of IL-1β (**c**), IL-6 (**d**) and TNF-α (**e**) and IL-10 (**f**) determined by qRT-PCR in the groups of Control, DNP, DNP + vehicle, SP, IL-35 and IL-35 + Ani. ^*^P < 0.05 vs. the control group; ^#^*P* < 0.05 vs. the DNP + vehicle group; ^$^*P* < 0.05 vs. the IL-35 group. *n* = 6 per group

**Fig. 3:**
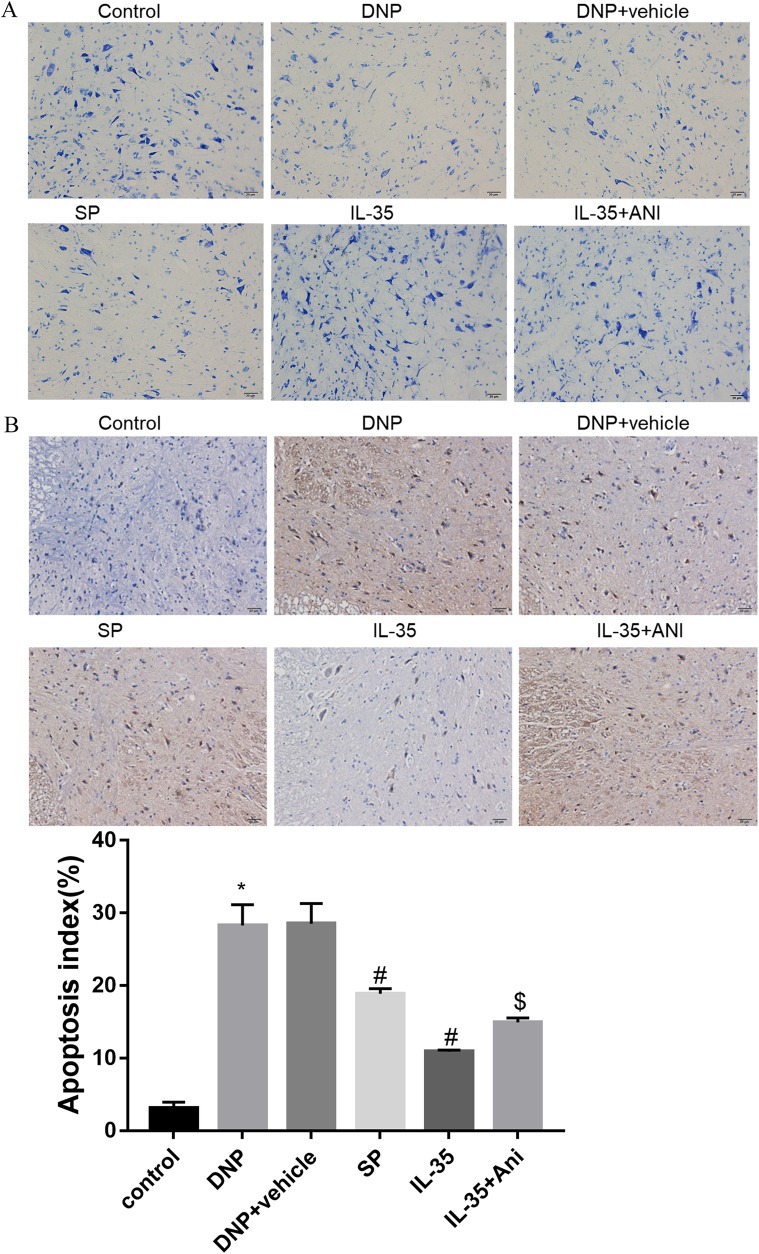
The anti-apoptotic effect of IL-35 in spinal cord tissues. Nissl staining to assess the loss of neurons (**a**) and spinal neuronal apoptosis detection using TUNEL assay (**b**) in Control, DNP, DNP + vehicle, SP, IL-35 and IL-35 + Ani groups, respectively. ^*^P < 0.05 vs. the control group; ^#^*P* < 0.05 vs. the DNP + vehicle group; ^$^*P* < 0.05 vs. the IL-35 group. n = 6 per group

Reports show that the protein levels of p-JNK increase in the spinal cord in a rat model of DNP. Therefore, we investigated whether IL-35 modulated inflammation in DNP progression via mediating JNK signaling. As expected, the protein levels of IL-35 in spinal cord were notably decreased in DNP and SP groups, but were increased in IL-35 and IL-35 + Ani groups compared to the control group (Fig. 2a; *P* < 0.05). We found that the protein levels of p-JNK were significantly increased in the spinal cord of DNP rats compared with the control group of rats. The 2 week treatment of IL-35 or SP600125 significantly inhibited the increase in the protein levels of p-JNK. Anisomycin further increased JNK phosphorylation (Fig. 2b; *P* < 0.05). Furthermore, qRT-PCR analysis showed that IL-35 or SP600125 injection could effectively exerted an inhibitory effect on IL-1β (Fig. 2c; *P* < 0.05), IL-6 (Fig. 2d; *P* < 0.05) and TNF-α (Fig. 2e; *P* < 0.05) secretion and IL-10 downregulation (Fig. 2f; *P* < 0.05) in the spinal cord induced by DNP. Activation of JNK by anisomycin further triggered the inflammatory response by upregulating IL-1β (Fig. 2c; *P* < 0.05), IL-6 (Fig. 2d; *P* < 0.05) and TNF-α (Fig. 2e; *P* < 0.05) and decreasing the mRNA levels of IL-10 (Fig. 2f; *P* < 0.05). Neuron survival in the spinal cord was further evaluated by Nissl staining. DNP group exhibited necrotic neurons and marked loss of Nissl granules in spinal cord tissues. Conversely, the injection of SP600125 or IL-35 recombinant protein markedly increased Nissl bodies, while anisomycin led to a decrease in the number of neurons and Nissl bodies (Fig. 3a). These results indicate that IL-35 administration might improve the histological changes of spinal cord and restore neuron function after DNP induction. Furthermore, the apoptotic cells in spinal cord tissues were significantly increased in the DNP group, while IL-35 or SP600125 injection reduced the number of TUNEL-positive cells, which were partly reversed by anisomycin (Fig. 3b; *P* < 0.05). Taken together, we concluded that IL-35 attenuated DNP through inhibiting neuroinflammation and neuroapoptosis by inhibition of JNK pathway.

## Discussion

DNP is a common diabetic complications resulting from damage or abnormal function of the central or peripheral nervous system. Mechanical, thermal, or chemical stimuli usually lead to allodynia and hyperalgesia, affecting patient’s quality of life [13]. The occurrence and development of DNP involves a variety of biochemical factors and alterations in anatomic structures in the peripheral nervous system and central nervous system [14]. Continuous hyperglycemia as the key initiating factor, could eventually provoke heightened excitability of peripheral nerve sensory and spinal dorsal horn neurons by controlling neuronal voltage-gated ion channels and affecting the production of nerve growth factor (NGF), leading to the spontaneous discharge of neurons and increased central sensitization, which is the basis of neuropathic pain [15, 16]. In addition, abundant studies provide strong evidence that the inflammatory microenvironment and the release of inflammatory mediators such as TNF-α are the precipitating factors for DNP development [17]. Rats injected with STZ displayed sensory dysfunction featured with allodynia and mechanical and thermal hyperalgesia, which is the most commonly used animal model to investigate the mechanisms of DNP [18]. In the present study, we demonstrated that changes of fasting insulin, the pain behavior and the expression changes of inflammatory cytokines (IL-1β, IL-6, TNF-α and IL-10) were observed in rats with DNP induced by STZ. Furthermore, the administration of IL-35 to DNP rats prevented the progression of inflammation by reducing the proinfammatory cytokines IL-1β, IL-6 and TNF-α levels and elevating anti-inflammatory cytokine IL-10 levels All these findings confirmed the protective effect of IL-35 against inflammatory response in STZ-induced DNP rats.

IL-35 is a characteristic immunosuppressive cytokine expressed by regulatory T cells (Tregs) [19]. Besides, it has been shown that IL-35 could be produced by regulatory B (Breg) cells [20, 21] and tolerogenic DCs (tolDCs) [22]. A growing number of evidence suggests that serum concentration of IL-35 was markedly lower in patients with type I or II diabetes [23, 24] and those with latent autoimmune diabetes mellitus in the adult (LADA) [25] compared to healthy controls. In recent years, several researches have proposed that IL-35 exhibits potent anti-inflammatory properties in the etiology and pathogenesis of inflammatory and autoimmune diseases. For instance, Zhou et al. [26] recently revealed that IL-35 was downregulated in serum and kidney tissues of STZ-induced diabetic rats. Additionally, Bettini et al. [27] reported that ectopic expression of IL-35 by pancreatic β-cells provided long-term protection against autoimmune diabetes via decreasing islet infiltration with substantial reductions in the number of CD4^+^ and CD8^+^ T cells. Recently, accumulating evidence has strongly implied that IL-35 administration reversed established hyperglycemia in nonobese diabetic (NOD) mouse model [23, 28]. Importantly, a recent study of Wang et al. [29] investigated the underlying role of IL-35 recombinant protein in colitis, and uncovered that IL-35 recombinant protein reverses inflammatory reaction by increasing the secretion of IL-10 and downregulating the expression of pro-inflammatory cytokines IL-6, TNF-α and IL-17 in acute colitis model. Interestingly, our study also found that intrathecal injection of IL-35 recombinant protein alleviated inflammation through inhibiting the expression of pro-inflammatory cytokines such as IL-1β, IL-6, and TNF-α, and promoting the secretion of IL-10 in rat serum and spinal cord of STZ-induced DNP rats.

Previous studies have demonstrated that nerve injury can lead to increased expression of the MAPK family members p38 MAPK, p-JNK, and p-ERK in dorsal root ganglion and spinal cord dorsal horn [18]. Specifically, elevated hippocampus expressions of p38 MAPK, p-JNK, and p-ERK were found in rats with diabetic neuropathy induced by STZ. Middlemas et al. [30] showed that JNK phosphorylation may convert the direct effects of raised glucose into impaired nerve conduction and neuropathic pain. In our study, both JNK inhibitor SP600125 and IL-35 decreased the expression levels of pro-inflammatory cytokines such as IL-1β, IL-6, and TNF-α, and spinal neuronal apoptosis and enhanced the expression of anti-inflammatory cytokine IL-10 in serum and spinal cord, while JNK activator anisomycin abolished the anti-inflammatory and anti-apoptotic effect of IL-35.

## Conclusion

This study reveals that IL-35 showed anti-inflammatory and anti-apoptotic properties in a STZ-induced DNP rat model via inhibiting JNK signaling pathway, highlighting IL-35 as a candidate target for DNP treatment.

## Data Availability

The datasets used and/or analyzed during the current study are available from the corresponding author on reasonable request.

## Abbreviations

Breg: regulatory B
DAB: Diaminobenzine
DMSO: Dimethylsulfoxide
DNP: Diabetic neuropathic pain
EBI3: Epstein-Barr virus-induced gene 3
ERK: Extracellular signal-regulated protein kinase
GDM: Gestational diabetes mellitus
IL: Interleukin
ISI: Insulin sensitivity index
JNK: c-Jun N-terminal kinase
LADA: Latent autoimmune diabetes mellitus in the adult
LPS: Lipopolysaccharide
MAPK: Mitogen-activated protein kinase
MWT: Mechanical withdrawal thresholds
NF-κB: nuclear factor
NGF: Nerve growth factor
NOD: Nonobese diabetic
PAGE: Polyacrylamide gel electrophoresis
PBS: Phosphate-buffered saline
PDR: Proliferative diabetic retinopathy
PE: Preeclampsia
PVDF: Polyvinylidene fluoride
qRT-PCR: quantitative reverse transcription PCR
SD: Standard deviation
STAT1: Signal transducer and activator of transcription 1
STZ: Streptozotocin
TBST: Tris buffered saline tween
TNF: Tumor necrosis factor
tolDCs: tolerogenic DCs
Tregs: regulatory T cells
TWL: Thermal withdrawl latency

## References

[1] Zimmet PZ (2017). Diabetes and its drivers: the largest epidemic in human history?. Clin Diabetes Endocrinol.

[2] Tesfaye S, Selvarajah D, Gandhi R, Greig M, Shillo P, Fang F, Wilkinson ID (2016). Diabetic peripheral neuropathy may not be as its name suggests: evidence from magnetic resonance imaging. Pain.

[3] Kiasalari Z, Rahmani T, Mahmoudi N, Baluchnejadmojarad T, Roghani M (2017). Diosgenin ameliorates development of neuropathic pain in diabetic rats: involvement of oxidative stress and inflammation. Retour Au Numéro.

[4] Lanna A, Gomes DC, Muller-Durovic B, Mcdonnell T, Escors D, Gilroy DW, Lee JH, Karin M, Akbar AN (2017). A sestrin-dependent Erk-Jnk-p38 MAPK activation complex inhibits immunity during aging. Nat Immunol.

[5] Wang D, Couture R, Hong Y (2014). Activated microglia in the spinal cord underlies diabetic neuropathic pain. Eur J Pharmacol.

[6] Sanna MD, Galeotti N (2018). The HDAC1/c-JUN complex is essential in the promotion of nerve injury-induced neuropathic pain through JNK signaling. Eur J Pharmacol.

[7] Zhang TT, Xue R, Fan SY, Fan QY, An L, Li J, Zhu L, Ran YH, Zhang LM, Zhong BH (2018). Ammoxetine attenuates diabetic neuropathic pain through inhibiting microglial activation and neuroinflammation in the spinal cord. J Neuroinflammation.

[8] Li J, Zhao PP, Hao T, Wang D, Wang Y, Zhu YZ, Wu YQ, Zhou CH (2017). Urotensin II inhibitor eases neuropathic pain by suppressing the JNK/NF-κB pathway. J Endocrinol.

[9] Yan A, You H, Zhang X (2018). Levels of interleukin 27 and interleukin 35 in the serum and vitreous of patients with proliferative diabetic retinopathy. Ocul Immunol Inflamm.

[10] Cao W, Wang X, Chen T, Xu W, Feng F, Zhao S, Wang Z, Hu Y, Xie B (2018). Maternal lipids, BMI and IL-17/IL-35 imbalance in concurrent gestational diabetes mellitus and preeclampsia. Exp Ther Med.

[11] Peng M, Wang Y, Qiang L, Xu Y, Li C, Li T, Zhou X, Xiao M, Wang J (2018). Interleukin-35 inhibits TNF-alpha-induced Osteoclastogenesis and promotes apoptosis via shifting the activation from TNF receptor-associated death domain (TRADD)-TRAF2 to TRADD-Fas-associated death domain by JAK1/STAT1. Front Immunol.

[12] Xiaojin S, Shu M, Xinyuan L, Hang X, Massimo M, Pascual DW, Huimin S, Xiaohua J, Hong W, Xiao-Feng Y (2015). Interleukin-35 inhibits endothelial cell activation by suppressing MAPK-AP-1 pathway. J Biol Chem.

[13] Shahid M, Subhan F, Ahmad N, Ali G, Akbar S, Fawad K, Sewell RD (2017). Topical gabapentin gel alleviates allodynia and hyperalgesia in the chronic sciatic nerve constriction injury neuropathic pain model. Eur J Pain.

[14] Hébert HL, Veluchamy A, Torrance N, Smith BH (2017). Risk factors for neuropathic pain in diabetes mellitus. Pain.

[15] Zhou Y, Wang XL, Yu HB (2017). Current status of ion channels as drug targets for diabetic neuropathic pain. Yao Xue Xue Bao.

[16] Velasco R, Navarro X, Gil-Gil M, Herrando-Grabulosa M, Calls A, Bruna J (2017). Neuropathic pain and nerve growth factor in chemotherapy-induced peripheral neuropathy: prospective clinical-pathological study. J Pain Symptom Manag.

[17] Leung L, Cahill CM (2010). TNF-alpha and neuropathic pain--a review. J Neuroinflammation.

[18] Jiang-Kun D, Yan W, Hong C, Bo M, Cong-Cong H, Guo C, Jun L, Xue-Jun S, Qing-Quan L (2014). Establishment of a rat model of type II diabetic neuropathic pain. Pain Med.

[19] Zongyi Y, Funian Z, Hao L, Xin W, Ying C, Jialin Z, Yongfeng L, Baifeng L (2017). Interleukin-35 mitigates the function of murine transplanted islet cells via regulation of Treg/Th17 ratio. PLoS One.

[20] Shen P, Roch T, Lampropoulou V, O'Connor RA, Stervbo U, Hilgenberg E, Ries S, Dang VD, Jaimes Y, Daridon C (2014). IL-35-producing B cells are critical regulators of immunity during autoimmune and infectious diseases. Nature.

[21] Wang RX, Yu CR, Dambuza IM, Mahdi RM, Dolinska MB, Sergeev YV, Wingfield PT, Kim SH, Egwuagu CE (2014). Interleukin-35 induces regulatory B cells that suppress autoimmune disease. Nat Med.

[22] Dixon KO, van der Kooij SW, Vignali DA, van Kooten C (2015). Human tolerogenic dendritic cells produce IL-35 in the absence of other IL-12 family members. Eur J Immunol.

[23] Singh K, Kadesjo E, Lindroos J, Hjort M, Lundberg M, Espes D, Carlsson PO, Sandler S, Thorvaldson L (2015). Interleukin-35 administration counteracts established murine type 1 diabetes--possible involvement of regulatory T cells. Sci Rep.

[24] Espes D, Singh K, Sandler S, Carlsson PO (2017). Increased Interleukin-35 levels in patients with type 1 diabetes with remaining C-peptide. Diabetes Care.

[25] Singh K, Martinell M, Luo Z, Espes D, Stalhammar J, Sandler S, Carlsson PO. Cellular immunological changes in patients with LADA are a mixture of those seen in patients with type 1 and type 2 diabetes. Clin Exp Immunol. 2019;197:64–73. 10.1111/cei.13289 PMC6591143 30843600

[26] Wenbin Z, Guojun G (2014). Resveratrol ameliorates diabetes-induced renal damage through regulating the expression of TGF-beta1, collagen IV and Th17/Treg-related cytokines in rats. West Indian Med J.

[27] Bettini M, Castellaw AH, Lennon GP, Burton AR, Vignali DA (2012). Prevention of autoimmune diabetes by ectopic pancreatic beta-cell expression of interleukin-35. Diabetes.

[28] Manzoor F, Johnson MC, Li C, Samulski RJ, Wang B, Tisch R (2017). beta-cell-specific IL-35 therapy suppresses ongoing autoimmune diabetes in NOD mice. Eur J Immunol.

[29] Wang Y, Mao Y, Zhang J, Shi G, Cheng L, Lin Y, Li Y, Zhang X, Zhang Y, Chen X (2018). IL-35 recombinant protein reverses inflammatory bowel disease and psoriasis through regulation of inflammatory cytokines and immune cells. J Cell Mol Med.

[30] Middlemas AB, Agthong S, Tomlinson DR (2006). Phosphorylation of c-Jun N-terminal kinase (JNK) in sensory neurones of diabetic rats, with possible effects on nerve conduction and neuropathic pain: prevention with an aldose reductase inhibitor. Diabetologia.

